# Impact of the HeartMate 3 continuous-flow left ventricular assist device in patients with small body size

**DOI:** 10.1093/icvts/ivac012

**Published:** 2022-02-01

**Authors:** Kohei Tonai, Satsuki Fukushima, Naoki Tadokoro, Satoshi Kainuma, Naonori Kawamoto, Takashi Kakuta, Ayumi Koga-Ikuta, Takuya Watanabe, Osamu Seguchi, Yasumasa Tsukamoto, Norihide Fukushima, Tomoyuki Fujita

**Affiliations:** 1 Department of Cardiovascular Surgery, National Cerebral and Cardiovascular Center, Suita, Osaka, Japan; 2 Department of Transplant Medicine, National Cerebral and Cardiovascular Center, Suita, Osaka, Japan

**Keywords:** Small patients, Ventricular assist device, Body surface area, HeartMate 3

## Abstract

**OBJECTIVES:**

Limited data are available for use of the HeartMate 3 (HM 3) left ventricular assist device in patients with a small body surface area (BSA). Because the HM 3 is currently the sole device available worldwide, we conducted a single-centre retrospective study of patients with a small BSA (<1.5 m^2^) who underwent HM 3 implantation to better understand the operative and postoperative management.

**METHODS:**

This study enrolled 64 consecutive patients who had undergone HM 3 implantation from August 2018 to July 2021. The patients were divided into 2 groups based on their BSA before the operation: BSA of <1.5 m^2^ (small BSA group, *n* = 18) and BSA of ≥1.5 m^2^ (regular BSA group, *n* = 46). The primary study endpoint was survival free of events such as disabling stroke and pump failure. The secondary endpoint was the frequency of adverse events.

**RESULTS:**

The average BSA was 1.38 m^2^ in the small BSA group. The overall event-free survival rate at 12 months was 100% and 86.7% in the small BSA group and regular BSA group, respectively, and no significant difference was found between the 2 groups (log-rank *P *=* *0.2). The number of cumulative adverse events of death, stroke of any severity, driveline infection, pump infection, ventricular arrhythmia, gastrointestinal Haemorrhage and pump failure was similar between the 2 groups.

**CONCLUSIONS:**

The HM 3 was safely implanted in patients with a small BSA, and postoperative outcomes were acceptable regardless of BSA. However, further research is needed to confirm the indications for HM 3 implantation in even smaller patients.

**Figure:**
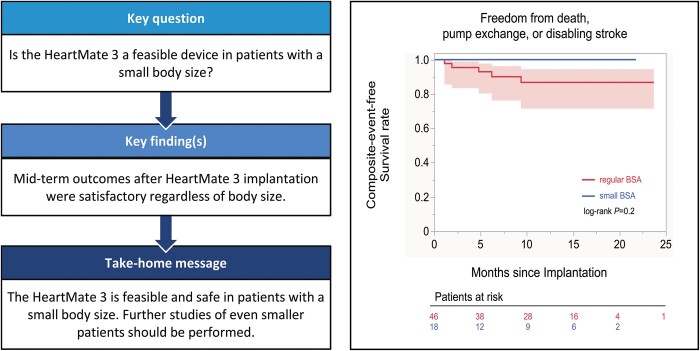

## INTRODUCTION

The HeartMate 3 (HM 3; Abbott, Chicago, IL, USA) left ventricular assist device (LVAD) is an essential treatment choice for a growing number of patients with end-stage advanced heart failure, either as a bridge to heart transplantation or as a destination therapy. Earlier generations of implantable pumps were initially designed for patients with a larger body surface area (BSA), typically those with a BSA of ∼2.0 m^2^ [1]. Patients with a smaller BSA (<1.5 m^2^) were usually excluded as candidates for implantable LVADs because of the bulky pump size of these devices. However, continuous upgrades in device technology and the transition from the HeartMate II to the HM 3 have facilitated the wider use of LVADs in patients with smaller body sizes [2–6]. More recently, the U.S. Food and Drug Administration expanded use of the HM 3 to paediatric patients, leading to an increase in the number of patients with a smaller BSA receiving LVADs [3]. A smaller pump size has significant consequences for Japanese and other Asian populations, which have an average BSA of ∼1.6 m^2^; in contrast, the average adult BSA in the Interagency Registry for Mechanically Assisted Circulatory Support (INTERMACS) registry is ∼2.1 m^2^. However, only a few studies have compared the clinical outcomes of durable LVAD therapy in smaller patients with those in a regular BSA population [2–5, 7, 8]. Therefore, this study was performed to review the outcomes and adverse events of HM 3 implantation in patients with a smaller BSA and to explore tips and pitfalls in managing such patients.

## MATERIALS AND METHODS

### Study design

This retrospective single-centre study included patients with end-stage heart failure who underwent implantation of the HM 3 device at the National Cerebral and Cardiovascular Center from August 2018 to July 2021. All patients were pathologically diagnosed based on biopsy specimens of the right ventricle before LVAD implantation. The surgical indication for durable LVAD implantation was discussed by the institutional multidisciplinary heart team. Data collection was performed in July 2021.

**Figure 1:**
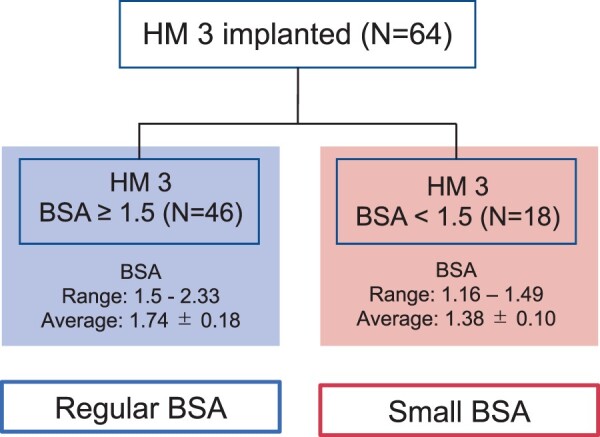
Categorization of all 64 patients who underwent implantation with a HeartMate 3 from August 2018 to July 2021. BSA: body surface area; HM 3: HeartMate 3.

Patients were divided into 2 groups based on pre-specified clinically important cut-off points for the BSA before implantation of the LVAD: BSA of <1.5 m^2^ (small BSA group, *n* = 18) and BSA of ≥1.5 m^2^ (regular BSA group, *n* = 46) (Fig. 1) [2, 4, 8].

The primary study endpoint was LVAD-related mortality after durable LVAD implantation and a composite of survival free of disabling stroke and survival free of reoperation to replace or remove a malfunctioning device. The secondary endpoint was the frequency of adverse events that were defined in the INTERMACS report [9].

### Ethics

Either the patients or their legal representatives preoperatively provided written informed consent for surgery and the use of their data for diagnostic and research purposes. The study was conducted in compliance with the Declaration of Helsinki, International Conference on Harmonization/Good Clinical Practice. The study was approved by the National Cerebral and Cardiovascular Center Institutional Review Board for Clinical Research (approval number: M30-026, approval date: 18 July 2018).

### Surgical procedure

All operations were performed following median sternotomy under mild hypothermic cardiopulmonary bypass. An appropriate pericardial incision was made to achieve an ideal inflow angle and pump stabilization. In particular, for smaller patients, a deeper pericardial incision was performed 3–4 cm above the phrenic nerve, and the costal attachments of the diaphragm were sometimes divided to create space for an outflow graft and the bend relief. In certain cases, the bend relief was affixed to the diaphragm using stitches to prevent pump migration. Polytetrafluoroethylene membranes were placed both between the pump and left lung as well as between the outflow graft and sternum to prevent adhesions.

Concomitant tricuspid annuloplasty was performed with the use of a prosthetic ring in patients with at least moderate tricuspid regurgitation (TR) preoperatively (*n* = 14, 22%). Aortic valve repair using Park’s stitch was performed in patients with mild or worse aortic insufficiency preoperatively (*n* = 11, 17%) [10]. In patients with poor right ventricular function who failed to wean off cardiopulmonary bypass under LVAD support, a right ventricular assist device was added following cannulations into the main pulmonary artery and right atrium via the femoral vein (*n* = 3, 5%).

### Perioperative medical treatment and laboratory examinations

Considering that early postoperative heparinization reduces haemolysis [11], continuous heparin infusion was started as soon as haemostasis was achieved. This infusion was titrated targeting an activated prothrombin time of 50–60 s until the international normalized ratio reached 2.0. A vitamin K antagonist was started on Day 1 or 2 postoperatively, targeting an international normalized ratio of 2.0–3.0. Aspirin (100 mg daily) was started when the platelet count in the blood was >100 000/mm^3^.

Transthoracic echocardiography (TTE) and a right heart catheter (RHC) study were performed preoperatively in all patients, and postoperative TTE was performed at 54.6 ± 26.7 days and an RHC study at 31.8 ± 9.1 days after LVAD implantation. In the RHC study, the right atrial pressure to pulmonary capillary wedge pressure ratio was calculated as an indicator of right ventricular dysfunction [12].

### Statistical analysis

All statistical analyses were performed using JMP software, ver. 16.0 (SAS Institute Inc., Cary, NC, USA). For comparison of the preoperative background factors and clinical characteristics of patients between the small BSA and regular BSA groups, continuous variables were compared using the unpaired *t-*test and categorical variables were compared using the chi-square test or Fisher’s exact test, as appropriate. Continuous variables are shown as mean ± standard deviation, and categorical variables are shown as number and percentage. To assess changes in TTE and RHC study variables before and after LVAD surgery, repeated-measures multivariate analysis of variance was used to calculate the interaction *P*-value. Overall survival and event-free survival were estimated using Kaplan–Meier curves and compared between groups using the log-rank test. Statistical significance was defined as *P *<* *0.05.

## RESULTS

### Patients’ characteristics and preoperative variables

The LVAD was implanted as a bridge to transplantation in 60 patients who were listed in the Organ Transplantation Network, Japan before surgery, whereas 4 patients underwent durable LVAD implantation as the destination therapy. Clinical follow-up was completed at the end of the study in all patients (100%), and the mean follow-up period was 318 ± 194 days.

The baseline characteristics of the patients in the 2 groups are summarized in Table 1. The mean BSA of the complete cohort was 1.64 ± 0.2 m^2^. No inter-group differences were observed in age, INTERMACS profile level or purpose of LVAD implantation, although female sex was more common in the smaller BSA patient population. Idiopathic dilated cardiomyopathy was the major aetiology of heart failure in both groups, whereas the proportion of patients with ischaemic cardiomyopathy was smaller in the small BSA group (Table 1).

**Table 1:** Baseline clinical characteristics on admission of total cohort and comparison between the regular BSA and small BSA groups

	Total cohort	Regular BSA	Small BSA	*P*-value
	(*n* = 64)	(*n* = 46)	(*n* = 18)	
Age, years	46.3 ± 14.7	48.7 ± 12.3	40 ± 18.5	0.0309
Male, *n* (%)	40 (62.5)	38 (82.6)	2 (11.1)	<0.0001
Body mass index, kg/m^2^	21.2 ± 3.5	18.6 ± 2.5	22.2 ± 3.4	0.0002
Body surface area, m^2^	1.64 ± 0.2	1.74 ± 0.18	1.38 ± 0.09	<0.0001
INTERMACS profile level				
1	21 (32.8)	15 (32.6)	6 (33.3)	0.62
2	23 (35.9)	18 (39.1)	5 (27.8)	
3	20 (31.3)	13 (28.3)	7 (38.9)	
Purpose of LVAD				
Bridge to transplant	60 (93.7)	44 (95.7)	16 (88.9)	0.34
Destination therapy	4 (6.3)	2 (4.3)	2 (11.1)	
Aetiology				
DCM	33 (51.5)	24 (52.1)	9 (50)	0.059
dHCM	6 (9.4)	5 (10.9)	1 (5.6)	
ICM	10 (15.6)	9 (19.6)	1 (5.6)	
Myocarditis	3 (4.7)	2 (4.4)	1 (5.6)	
Others	12 (18.8)	6 (13)	6 (33.3)	
Valvular disease				
Moderate or severe TR	5 (7.8)	4 (8.7)	1 (5.6)	0.66
Moderate or severe MR	11 (17.2)	8 (17.4)	3 (16.7)	0.94
Moderate or severe AI	0 (0)	0 (0)	0 (0)	

AI: aortic insufficiency; DCM: dilated cardiomyopathy; dHCM: dilated phase of hypertrophic cardiomyopathy; ICM: ischaemic cardiomyopathy; INTERMACS: Interagency Registry for Mechanically Assisted Circulatory Support; LVAD: left ventricular assist device; MR: mitral regurgitation; TR: tricuspid regurgitation; VAD: ventricular assist device.

The preoperative and postoperative TTE and RHC study results are summarized in Table 2. The preoperative left ventricular end-diastolic and systolic diameter in the small BSA group were 55.1 and 49.5 mm, respectively, and these were significantly smaller than those in the regular BSA group (*P *=* *0.0027 and 0.0034, respectively). Furthermore, the postoperative left ventricular end-diastolic and systolic diameter in the small BSA group were 49.2 and 38.5 mm, respectively, and these were significantly smaller than those in the regular BSA group (*P *=* *0.019 and 0.037, respectively). The left ventricular end-diastolic volume estimated using the Teichholz formula was reduced after LVAD implantation in both groups (reduction volume of 99 ± 93 ml in the regular BSA group and 87 ± 76 ml in the small BSA group, interaction *P *=* *0.7).

**Table 2:** Comparison of preoperative and postoperative variables between the regular BSA and small BSA groups and interaction *P*-value for each variable

	Preoperative			Postoperative			
	Regular BSA	Small BSA	*P-*value	Regular BSA	Small BSA	*P*-value	Interaction *P*-value
	(*n* = 46)	(*n* = 18)		(*n* = 46)	(*n* = 18)		
TTE							
LVEDD, mm	68.4 ± 14.1	55.1 ± 14.3	0.0027	54.5 ± 14.6	43.2 ± 8.2	0.019	0.98
LVESD, mm	62.6 ± 14.5	49.5 ± 14.2	0.0034	49.2 ± 15.6	38.5 ± 9.4	0.037	0.92
LVEF, %	20.1 ± 10.4	24.1 ± 2.6	0.19	19.3 ± 8.2	24 ± 8.4	0.13	0.6
eLVEDV, ml	256 ± 118	161 ± 87	0.0061	159 ± 97	88 ± 38	0.024	0.7
RHC							
SABP, mmHg	88.7 ± 11.3	89.1 ± 18.9	0.93	84.8 ± 10.5	80.1 ± 4.9	0.21	0.69
CI, l/min/m^2^	2.1 ± 0.6	1.7 ± 0.5	0.03	2.7 ± 0.05	2.4 ± 0.08	0.0084	0.66
PCWP, mmHg	16.8 ± 8.9	15.1 ± 6.6	0.51	5.8 ± 3.4	5 ± 2.6	0.48	0.93
MPAP, mmHg	24.4 ± 11.8	22.8 ± 7.1	0.63	13.7 ± 4.4	13 ± 2.2	0.62	0.76
PVR, Wood units	2.2 ± 1.2	3.3 ± 1.4	0.011	1.7 ± 0.6	2.4 ± 0.7	0.0026	0.56
RAP, mmHg	7.4 ± 6.0	6.7 ± 4.0	0.7	5.3 ± 4.1	5.8 ± 3.8	0.73	0.55
RA/PCWP ratio	0.6 ± 0.7	0.6 ± 0.5	0.92	0.9 ± 0.6	1.2 ± 0.5	0.24	0.29

CI: cardiac index; eLVEDV: estimated left ventricular end-diastolic volume; LVEDD: left ventricular end-diastolic diameter; LVEF: left ventricular ejection fraction; LVESD: left ventricular end-systolic diameter; MPAP: mean pulmonary artery pressure; PCWP: pulmonary capillary wedge pressure; PVR: pulmonary vascular resistance; RAP: right atrial pressure; RHC: right heart catheter; SABP: systolic arterial blood pressure; TTE: transthoracic echocardiography.

Pulmonary vascular resistance was significantly higher preoperatively in the small BSA group, although the right atrial pressure to pulmonary capillary wedge pressure ratio was similar in the 2 groups. However, the degree of change in all RHC study variables was not significantly different between the 2 groups (interaction *P *>* *0.05 for all).

### Pump speed settings

**Figure 2:**
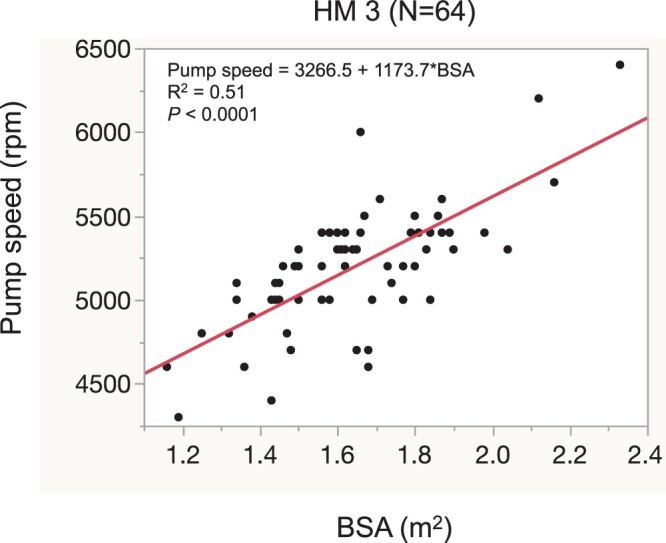
Correlation between body surface area and pump speed setting at intensive care unit entry for each device. BSA: body surface area; HM 3: HeartMate 3.

The pump speed settings were perioperatively adjusted according to transoesophageal echocardiography and RHC study findings. The pump speed settings (rpm) on intensive care unit entry for HM 3 implantation were plotted (Fig. 2); the pump speed linearly increased in correlation with the increase in the BSA (*P *<* *0.0001).

### Operation and postoperative course

The operation time, concomitant procedures, rate of perioperative right ventricular assist device requirement and length of intensive care unit stay were not different between the 2 groups (Table 3).

**Table 3:** Comparison of perioperative and postoperative variables between the regular BSA and small BSA groups

	Regular BSA	Small BSA	*P*-value
	(*n* = 46)	(*n* = 18)	
Operation time, min	289 ± 153	256 ± 67	0.38
Concomitant procedures			
TAP for TR, *n* (%)	10 (21.7)	4 (22.2)	0.97
AVP for AI, *n* (%)	8 (17.4)	3 (16.7)	0.94
RVAD requirement, *n* (%)	3 (6.5)	0 (0)	0.15
Postoperative intubation, hours	15.9 ± 17.5	9.3 ± 6.2	0.12
ICU stay, days	5.7 ± 9.2	6.8 ± 3.8	0.67

AI: aortic insufficiency; AVP: aortic valve plasty; ICU: intensive care unit; RVAD: right ventricular assist device; TAP: tricuspid valve plasty; TR: tricuspid regurgitation.

### Survival and freedom from death, disabling stroke and pump failure

**Figure 3:**
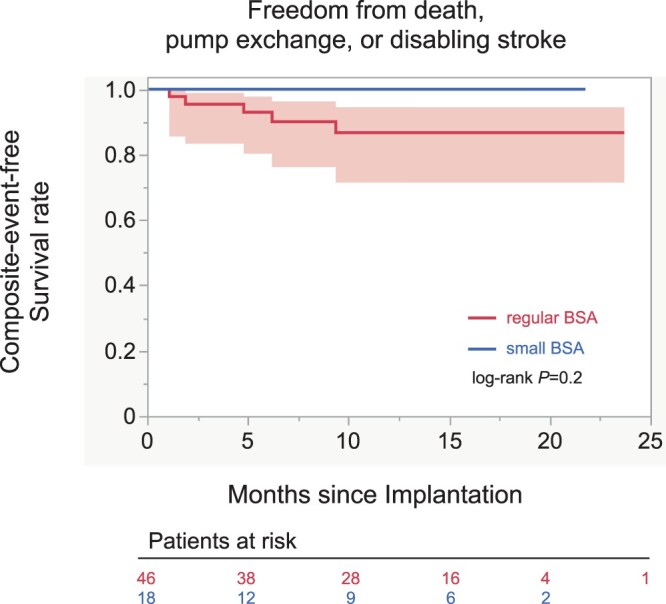
Comparison of survival rate free from pump exchange or disabling stroke between the regular and small BSA groups in the Kaplan–Meier analysis. BSA: body surface area.

There were no cases of 30-day mortality in either group. During the follow-up, 4 patients (8.7%) died of either stroke (*n* = 2) or sepsis (*n* = 2) in the regular BSA group, and no patients died in the small BSA group (Table 4). The 6-month and 1-year freedom from composite adverse events, defined as death, disabling stroke or pump failure, were 100% and 100% in the small BSA group and 92.9% and 86.7% in the regular BSA group, respectively. No significant difference was observed between the 2 study groups (log-rank *P *=* *0.2; Fig. 3).

**Table 4:** Comparison of mortality, fatal events and cumulative adverse events between the regular BSA and small BSA groups

	Regular BSA	Small BSA	*P*-value
	(*n* = 46)	(*n* = 18)	
Death	4 (8.7)	0 (0)	0.097
Death, pump exchange and disabling stroke	5 (10.9)	0 (0)	0.063
Adverse events			
Driveline infection	5 (10.9)	1 (5.6)	0.75
Pump infection	0 (0)	0 (0)	
Cerebrovascular accidents	1 (2.2)	0 (0)	0.41
Gastrointestinal bleeding	1 (2.2)	0 (0)	0.48
Ventricular arrhythmia	2 (4.3)	0 (0)	0.33
Right ventricular failure	7 (18.4)	0 (0)	0.029
Pump thrombus	0 (0)	0 (0)	

BSA: body surface area.

### Adverse events

Accumulative adverse events throughout the follow-up period are shown in Table 4. There was no significant inter-group difference in accumulative adverse events with the exception of right ventricular failure, which was more common in the regular BSA group (*P *=* *0.029).

## DISCUSSION

This study compared the clinical outcomes of patients with a BSA of <1.5 m^2^ who underwent implantation of the HM 3 with those of patients with a BSA of ≥1.5 m^2^. Composite event-free survival was not significantly different between the 2 groups. The postoperative cumulative incidence of all adverse events was also not significantly different between the 2 groups with the exception of right ventricular failure.

Consistent with previous reports, pump speeds tended to be set lower to meet the lesser circulatory demands in patients with a small BSA (Fig. 2) [4, 8]. Excessively high pump speeds may emphasize right heart failure, an independent risk factor for worse mid-term outcomes, by causing interventricular septal deviation towards the left [13]; this occurs more frequently in patients with a small BSA because of the lesser flow demand [4, 8]. Because lower flow with an LVAD is a risk factor for thrombus formation, which may result in stroke or bleeding [4, 7], consistent pump speed adjustment with haemodynamic monitoring using RHC studies and echocardiography to maintain the left ventricular geometry is required in patients with a small BSA. This resulted in the lack of significant differences in the stroke rate between the 2 groups.

In patients with a small left ventricular size (<55 mm), which is also an independent risk factor for worse outcomes [14], intraoperative adjustment of the angle of the inflow cannula is key. To adjust the angle of the inflow cannula in the limited thoracic space, a deep pericardial incision and partial division of the costal attachment of the diaphragm are helpful to place the pump precisely under the guidance of intraoperative transoesophageal echocardiography [15, 16]. Despite left ventricular volume reduction after LVAD implantation, the intrapericardial space is limited in patients with a smaller BSA, making it difficult to secure sufficient intrapericardial space for a pump (80-ml HM 3 displacement volume). Therefore, all patients underwent computed tomography to evaluate whether adequate space was present and to assess the need for extrapericardial placement. A recent report indicated that 3-dimensional computed tomography is useful for finding space for the pump and simulating insertion of the LVAD [17]. This should be considered when renal function allows.

Regardless of body size, addressing TR to ensure optimal long-term outcomes is important because TR exacerbates right heart failure [18]. Especially, in our patients with a small BSA, perioperative pulmonary vascular resistance was significantly higher than that in patients with a regular BSA; this means that right heart failure could easily occur in smaller patients. Right heart failure is one of the major causes of rehospitalization and mortality. In our cohort, 14 patients (30.4%) underwent concomitant tricuspid annuloplasty. This number was higher than in a previous report [19]. Hence, tricuspid annuloplasty should be considered when the TR is greater than mild or when tricuspid annular dilatation is found because tricuspid annuloplasty can be performed without increasing operative mortality. The concomitant control of TR may have contributed to the favourable event-free survival in our cohort.

In this report, the smallest BSA was 1.16 m^2^. In a prior study, a patient with a BSA of 0.78 m^2^ was reported as the smallest patient to successfully undergo HM 3 implantation [3]; however, further evidence of the safety of implantation of an HM 3 for patients with a BSA <1.0 m^2^ is still required. We should carefully consider and evaluate the ability of the HM 3 to treat the patient population in which the HeartWare HVAD (Medtronic, Framingham, MA, USA) has previously been used [5].

### Limitations

The current study is limited by its retrospective, observational, single-centre design and small Japanese cohort. In this study, we conducted many tests without adjustment for multiple testing. Therefore, the *P*-value may be interpreted as not confirmatory but rather descriptive. The operative techniques of LVAD implantation for patients with small body sizes established in this report have been constantly refined through the accumulation of such cases and were not planned. Furthermore, baseline differences in background aetiologies, the severity of heart failure, and the degree of underlying right ventricular function were not considered when comparing the outcomes between the regular and small BSA groups.

## CONCLUSION

The HM 3 was safely implanted in patients with a small BSA, and the postoperative outcomes were acceptable regardless of the BSA. However, further research is needed to confirm the indications for HM 3 implantation in even smaller patients.

## Notes

Presented at the 35th Annual Meeting of the European Association for Cardio-Thoracic Surgery, Barcelona, Spain, 13–16 October 2021.

## ABBREVIATIONS

BSA: Body surface area
HM 3: HeartMate 3
LVAD: Left ventricular assist device
INTERMACS: Interagency Registry for Mechanically Assisted Circulatory Support
TR: Tricuspid regurgitation
TTE: Transthoracic echocardiography
RHC: Right heart catheter
